# A suggested data structure for transparent and repeatable reporting of bibliographic searching

**DOI:** 10.1002/cl2.1288

**Published:** 2022-11-23

**Authors:** Neal R. Haddaway, Melissa L. Rethlefsen, Melinda Davies, Julie Glanville, Bethany McGowan, Kate Nyhan, Sarah Young

**Affiliations:** ^1^ Leibniz‐Centre for Agricultural Landscape Research (ZALF) Müncheberg Germany; ^2^ Africa Centre for Evidence University of Johannesburg Johannesburg South Africa; ^3^ Health Sciences Library & Informatics Center University of New Mexico Albuquerque New Mexico USA; ^4^ Kaiser Permanente Center for Health Research Portland Oregon USA; ^5^ Glanville.info York United Kingdom; ^6^ Libraries and School of Information Studies Purdue University West Lafayette Indiana USA; ^7^ Harvey Cushing/John Hay Whitney Medical Library Yale University New Haven Connecticut USA; ^8^ Environmental Health Sciences, Yale School of Public Health Yale University New Haven Connecticut USA; ^9^ University Libraries Carnegie Mellon University Pittsburgh Pennsylvania USA

## Abstract

Academic searching is integral to research activities: (1) searching to retrieve specific information, (2) to expand our knowledge iteratively, (3) and to collate a representative and unbiased selection of the literature. Rigorous searching methods are vital for reliable, repeatable and unbiased searches needed for these second and third forms of searches (exploratory and systematic searching, respectively) that form a core part of evidence syntheses. Despite the broad awareness of the importance of transparency in reporting search activities in evidence syntheses, the importance of searching has been highlighted only recently and has been the explicit focus of reporting guidance (PRISMA‐S). Ensuring bibliographic searches are reported in a way that is transparent enough to allow for full repeatability or evaluation is challenging for a number of reasons. Here, we detail these reasons and provide for the first time a standardised data structure for transparent and comprehensive reporting of search histories. This data structure was produced by a group of international experts in informatics and library sciences. We explain how the data structure was produced and describe its components in detail. We also demonstrate its practical applicability in tools designed to support literature review authors and explain how it can help to improve interoperability across tools used to manage literature reviews. We call on the research community and developers of reference and review management tools to embrace the data structure to facilitate adequate reporting of academic searching in an effort to raise the standard of evidence syntheses globally.

## 1 BACKGROUND

Searching for information is an integral part of scientific research activities. We search for a number of different reasons. Firstly, we search to retrieve specific information we previously found and/or know is available. This may be new information that we are relatively certain exists in a specific place (e.g., a thesaurus, glossary or encyclopaedia), or it may be information we have previously found and need to relocate. Such searching is referred to as ‘lookup searching’ (Gusenbauer & Haddaway, 2020).

Secondly, we search to incrementally expand our knowledge on a topic by finding the most relevant result that is likely to match well with our needs, following trails of information to help us build an internal conceptual model of a topic. This form of searching is termed ‘exploratory searching’ (Gusenbauer & Haddaway, 2020).

Thirdly, we may have developed a conceptual model of a topic and know what we want to search for, but wish to search in an unbiased, procedural way to obtain a potentially relevant evidence base that we can then read and screen for relevant information. This type of searching is referred to as ‘systematic searching’ (Gusenbauer & Haddaway, 2020; Jansen & Rieh, 2010) and is integral to systematic reviews and evidence‐informed decision‐making that aim to summarise large bodies of evidence in a reliable and robust way.

Exploratory searching is vital for planning systematic searches, and for conducting scoping reviews and other forms of syntheses (bringing together scientific information) that aim to improve understanding of the nature of an evidence base. For both exploratory and systematic searching, researchers often want/need to show the methods they used to search for information. This is so that they can demonstrate any efforts to reduce bias and increase comprehensiveness in their results. This transparency and the resultant repeatability are integral to robust evidence syntheses (Lefebvre et al., 2019; Page et al., 2021) and evidence‐informed decision making (Eden et al., 2011).

Despite the broad awareness of the importance of transparency in reporting search activities in evidence syntheses, such as systematic reviews and systematic maps (Koffel & Rethlefsen, 2016; Maggio et al., 2011; Mullins et al., 2014; Rader et al., 2014; Yoshii et al., 2009), it is only recently that the importance of searching has been highlighted and made the explicit focus of reporting guidance (PRISMA‐S; Rethlefsen et al., 2021). Previous efforts focusing on transparency in reporting systematic reviews had only limited focus on the details of searching (PRISMA 2009; (Moher et al., 2009)), and such details were far from allowing full repeatability. This lack of search history transparency in evidence syntheses prevents full assessment of the quality of the searches: the inclusion of librarians as co‐authors in systematic reviews has been shown to be correlated with higher quality searching (Rethlefsen et al., 2015; Schellinger et al., 2021), but a lack of detail prevents any assessment of conduct quality.

Robust exploratory and systematic searches typically involve searches for both traditional academic information and grey literature (non‐commercially produced reports and papers, (Eden et al., 2011; Higgins et al., 2018; Kugley et al., 2017; Schöpfel & Farace, 2010). Searches for academic information typically revolve around searches of bibliographic databases (defined as a data set of bibliographic information that, for a given search strategy on a given date and time, would return a fixed and identical set of results) (Eden et al., 2011; Higgins et al., 2018), such as Scopus (http://www.scopus.com), but also often involve a suite of alternative sources and methods, including citation searching (Wright et al., 2014). Grey literature searches are, by their very nature, diverse and highly topic specific: typically the websites of tens of organisations and other repositories are manually searched for potentially relevant documents, with idiosyncratic/varied procedures that are not easily reported in a standardised format (Canadian Agency for Drugs and Technologies in Health, 2018). Academic searches of bibliographic databases, however, are far more consistent. Despite this, to date there have been only limited attempts to provide a standard way of reporting searches of bibliographic databases (e.g., Gulhane, 2009; Bethel et al., 2021; de Jonge & Lein, 2015; Lyon et al., 2014).

**Figure 1:**
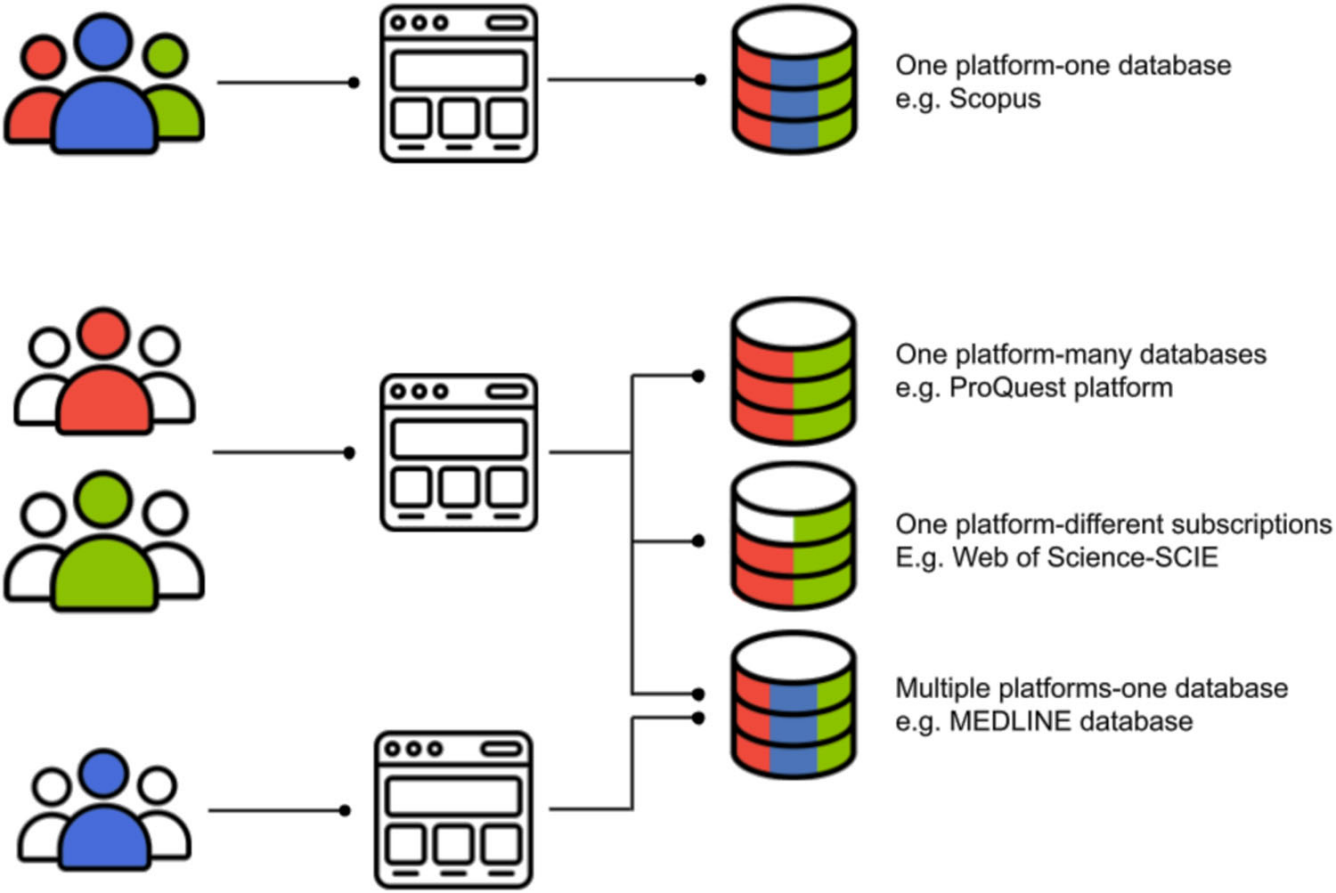
Schematic demonstrating the difference between platforms and databases, highlighting that different institutions may also subscribe to different extents of individual databases. The people on the left of the image represent different institutional subscriptions: the central square icons represent access platforms: the columns represent the year ranges (volume in colour) available for each database according to the users' institutional subscription (colours). Multi‐coloured columns indicate databases accessible through different institutional subscriptions. Platforms may provide access to multiple databases. Different date ranges for a database may be provided to different institutional subscriptions or via different platforms. Some databases may be accessible via multiple platforms.

Ensuring bibliographic searches are reported in a way that is transparent enough to allow for full repeatability or evaluation is not easy. There are a variety of reasons transparent, repeatable searches are challenging to ensure:

1. Researchers use both multi‐database search platforms and individual databases. These systems differ in how the database (i.e., the specific, incrementally updated collection of bibliographic data) is searched (occasionally simultaneously in combination with other databases) and how the platform providing the search facility performs the search (see Figure 1). *This diversity in search systems causes widespread misunderstandings regarding what is a database* (i.e., what is a repeatable single resource). For example, many researchers believe that Web of Science is a database or that Web of Science Core Collection is a set of fixed databases. In fact, Web of Science is a platform through which many different databases can be searched, whilst Web of Science Core Collection is a set of between one and seven databases (Clarivate, 2022), and the time spans available to any user depend on their institutional subscription. Web of Science Core Collection is therefore not a repeatable single database, although this is often referred to as such in systematic reviews (Liu, 2019).
2. In part, because of the diversity in search systems described above, but also because of the complex nature of bibliographic searching, *it is not immediately clear what information is sufficient for repeatability*. Many search settings are set by default (e.g., lemmatization and stemming), but many others must be selected (e.g., date restrictions). There are further settings which are established at a subscription level, and users may be unaware of them (e.g., the default Boolean/Phrase search mode in EBSCO is customisable by a host institution [EBSCO Industries, 2022]). As a result, many systematic review authors do not provide sufficient information to allow the searches to be precisely repeated.
3. Broadly speaking, *there is a lack of awareness, use, and enforcement of reporting standards in systematic reviews*. This applies to all aspects of review methods, but is particularly evident in search methods (de Kock et al., 2021; Page et al., 2016; Sargeant et al., 2021). The PRISMA reporting standards (Page et al., 2021) have been supplemented recently by the PRISMA‐S extension for reporting searches (Rethlefsen et al., 2021), but these standards are not adopted by all journals, and are rarely enforced (Koffel & Rethlefsen, 2016; Nascimento et al., 2020; Tam et al., 2017).
4. Search strings (the collections of terms entered together into search facilities) are often long and complex, and *it is easy for authors to introduce errors when they report their search strings and full search strategies* if they transcribe text. Copying and pasting directly is less error prone, but not infallible. No standard file type exists for reporting search histories, so these data must typically be manually collated and reported in a review—a process that is at high risk of induced transcription errors (Sampson & McGowan, 2006).
5. Review authors must select from a myriad of possible places and ways to store search histories (and also search results) during conduct and when reporting their methods. In our experience, authors use a variety of tools to develop and track search histories including text document files (e.g., in Microsoft Word), spreadsheets (e.g., Google Sheets), digital notebooks (e.g., Microsoft OneNote), search history files exported from platforms (e.g., Web of Science), and review management tools (e.g., EPPI‐Reviewer). As mentioned above, *none of these search history storage systems uses a standard data format, making reporting complicated and unclear, and providing no means of interoperability*. Search history data cannot be exported from one system and uploaded to another without manual transcription or copying‐and‐pasting, which are error prone (see point 4 above).
6. The many bibliographic databases and platforms used to search them are constantly subject to changes and updates. Some of these changes only affect the search interface, but *changes to search systems regularly occur that provide different results* (Burns et al., 2021; García‐Puente et al., 2020; Sisson & Ouellette, 2021): for example, due to a change in a default setting.
7. Many review authors report their search histories in the supplementary information of the final published review manuscript. However, *storing search histories in journal supplementary files is not an ideal means of storing information* because: they are not typically peer‐reviewed or checked before publication; they are typically not protected by a guarantee to be archived permanently; there is no requirement for ensuring text is digitised (text files are sometimes converted to flat images that cannot be searched or copied, and digital PDFs may be poorly digitised); they are not discoverable independently (i.e., each file is not indexed in search engines such as Google Scholar as a separate entity), so may be particularly hard to find. Furthermore, interlibrary loans (ILL) do not cover provision of supplementary files, making them yet more inaccessible to many readers.

Because of the need to transcribe or copy‐and‐paste searches into bibliographic databases, the repeatability of searches is limited by reporting accuracy (*how correct*) and precision (*how rich*), and is further hampered by the degree of *digitisation* and *digital accessibility* (i.e., how easily text/data can be extracted and reused without the need for transcription).

In sum, the systems used by review authors to store and report their bibliographic searches are not designed for transparent and repeatable reporting. This could be remedied by establishing a standard data format for reporting bibliographic search activities: this standard should specify *what* information should be reported (e.g., which data fields), and *how* it should be formatted (to allow for digitisation and unambiguous human/machine readability).

Going beyond this, a standard file type could be developed that would allow search history information to be readily and efficiently transferred from one search system to another (e.g., from Scopus and PubMed), between different review management and reporting tools (e.g., EPPI Reviewer and Rayyan): this in turn would facilitate repeatability (allowing a third party to repeat and/or evaluate the original searches precisely). Such *interoperability* goes beyond tools that translate copied‐and‐pasted search strings between databases (e.g., Polyglot; http://sr-accelerator.com/#/polyglot) and allow for search histories to be sent from one database to another with no need for manual intervention or curation of the data itself.

We believe there are a suite of significant benefits from such a standardised file type for reporting search strategies. Firstly, it would allow the development of search archives that transparently store searches in publicly accessible and searchable repositories. The records in such a repository could be readily reused, evaluated, incrementally developed or amended, and cited, reducing research waste and improving research efficiency. In this way, search records could be open to public scrutiny and constructive feedback, further providing opportunities for improvement and learning. Secondly, this would support more complete reporting of search activities in evidence syntheses by setting expectations of which data fields to report. Thirdly, it would support interoperability and reusability of searches. Fourthly, it would facilitate evaluation and verification of search activities and error checking before, during and after searches and protocol/review publication. Fifthly, it could facilitate the creation of validated search filters/hedges, by supporting repositories of standard searches that could be incrementally refined. Finally, we believe that such repositories would allow for improved crediting for search specialists involved in designing and conducting searches by creating citable records that can be used to demonstrate impact.

### 1.1 Objectives

Here, we present a suggested data structure that reports all details necessary to allow full repeatability of bibliographic database searches. The data structure was produced collaboratively by a group of specialists in information and library science and evidence synthesis methodology. This data structure outlines what information should be reported, how it should be presented, and suggests a way that this information can be encoded in a data file that would facilitate digital evaluation, reuse and interoperability. We believe this data structure would be of greatest use to developers producing review management tools and search history repositories, but also to keen systematic review authors wishing to ensure their methods are reported to a high level of detail.

## 2 METHODS

We sought to assemble a diverse group of international experts from a range of professional backgrounds. We identified 19 experts and invited them to join the Advisory Group: 16 people responded positively and joined an online workshop introducing the project and its aims.

The Advisory Group was invited to comment on a draft data structure that had been prepared by NRH and MLR using a Google form. The draft structure consisted of five columns: item name; data example; textual description; requirement (compulsory or optional), and notes/comments. Members of the Advisory Group were asked to provide comments as one of three types: amendments to existing text; addition of items; exclusion of items.

The feedback was collated and the draft data structure adjusted accordingly. We present here the final proposed data structure, with the modifications following feedback from the Advisory Group described in detail in Supporting Information: File 1.

## 3 RESULTS AND DISCUSSION

### 3.1 The data structure

The draft data structure is presented in Table 1. Each item is accompanied by an example provided in JSON format (a text file format that is readable by humans and machines, providing a nested and hierarchical structure beyond what is possible with flat spreadsheets), a description, optional/compulsory status, and comments.

**Table 1:** Suggested fields/items, examples, descriptions, suggested optionality and notes of contents for a JSON file type for bibliographic search histories

Item	Example	Description	Compulsory/optional	Notes
Authors	“name”: “Seedre, M”	Identifying information for all authors (name is compulsory, ORCID and email are optional)	Compulsory	Names compulsory, email and ORDIC identifiers optional
	“ORCID”: “0000‐0002‐3635‐6354”			
	“email”: “m.seedre@mail.com”			
	“name”: “Felton, A”			
	“name”: “Lindbladh, M”			
Data entry date	“data_entry:” “2021.03.02”	Date the search strategy was deposited in the archive (YYYY.MM.DD)	Compulsory	Can be populated automatically on entry storage
Strategy export date	“strategy_exported”: “2021.03.02”	Date the search strategy was extracted from the platform. Predominantly just relevant for automatically exported search histories (YYYY.MM.DD)	Optional (unless exported directly)	Can be populated automatically if export date provided in search history file
Search conduct date	“search_conducted:” “2020.01.01”	Date the search was conducted (YYYY.MM.DD)	Optional (unless linked to a DOI (protocol or final review))	Can be populated automatically if export contains search conduct date in search history file
Search update date(s)	“search_updated_1” “2021.02.01”	Date(s) the search was updated (if applicable) (YYYY.MM.DD)	Optional	
String name	“final”	Short descriptive tag given to the parent or child strings for internal purposes	Optional	For example, ‘intervention’ for a substring composed of intervention terms
Keywords	“Alternative forest management”, “Even‐aged silviculture”, “Uneven‐aged silviculture”, “Partial harvest”, “Selection harvest”, “Clear cut”, “Clear fell”	Optional keywords (comma separated), eg from the review protocol or final report. Additional terms used to find the search string	Optional	
Related search records	“10.5438/67dgh-t5666oop”	DOI for any child entries, for example for specific databases or search blocks	Optional	Only available if this is a parent entry
Overview entry for this substring	“10.5438/67dgh-t56-78p”	DOI for the parent entry, for example the overarching search strategy	Optional	Only available if this is a child entry
Influential search histories (references)	“10.1079/searchRxiv/20210426080”	DOI(s) for any records that were influential in developing this search (not including child/parent records)	Optional	
Platform	“Web of Science”	Platform the database was accessed through	Compulsory	Can be populated from a list of databases/providers, or automatically if search history file exported. Only available if this is a single database entry or child entry
Database	“Social Sciences Citation Index (SSCI)”	Database/index name	Compulsory	Can be populated from a list of providers/platforms, or automatically if search history file exported. Only available if this is a single database entry or child entry
Search string/strategy	“TS=(clear‐cut* OR clearcut* OR clearfell* OR clear‐fell* OR “clear fell*” OR even‐aged OR uneven‐aged) AND (forest* OR tree*)”	Search string/strategy. Can be either provided just as a complete string as entered into a platform's search function or supplemented by following the full string with a JSON format character string designating a series of named search blocks (see the example).	Compulsory	Can be populated automatically from search history export
	“management block”: “clear‐cut* OR clearcut* OR clearfell* OR clear‐fell* OR “clear fell*” OR even‐aged OR uneven‐aged)”			
	“forest block”: “forest* OR tree*”			
Search fields	“TS”	Field(s) searched	Optional	Can be populated automatically from search history export. Optional if this is specified in the search string itself.
Database time coverage from	“1945.01.01”	First accessible date within the database (YYYY.MM.DD)	Optional	State ‘unknown’ if not known. Can be populated automatically from search history export
Database time coverage to	“2021.12.31”	Last accessible date within the database (YYYY.MM.DD)	Optional	State ‘unknown’ if not known. Can be populated automatically from search history export
Date limitations	“time_span:” “null”	Time span limitations applied	Optional	Can be populated automatically from search history export
Language	“language:” “en”	Language limitations applied	Optional	Can be populated automatically from search history export
Settings	“lemmatization:” “on”,	Other settings, including content restrictions (e.g., excluding conference proceedings)	Optional	Can be populated automatically from search history export
	“spellchecking:” “Suggest”,			
Quality assurance	“appears in published protocol”	Optional categorical description of the type of search: “exploratory search”, “appears in published protocol”, “appears in published review”, “peer‐reviewed”, “validated against benchmark articles” (multiple choices acceptable)	Optional	Multiple choice
Validation report	“10.5438/67dgh-t56-78b”	Optional DOI, citation, or URL for manuscript describing validation of the search string	Optional	
Peer review	“10.5438/67dgh-t56-78b”	Optional URL of public peer‐review comments on the search strategy	Optional	
Description	“The search was conducted as part of a systematic review, developed during the publication of a protocol ([link]) and finalised during conduct of a systematic review ([link])”	Optional description of the context of the search	Optional	
Review question	“What is the impact of continuous cover forestry compared to clearcut forestry on stand‐level biodiversity in boreal and temperate forests?“	The review question of focus for the search strategy	Optional	
Review type	“Systematic review”	Optional, one of the following: “systematic review”, “systematic map”, “scoping review”, “rapid review”, “other literature review”		
Linked review protocol/registered report	“10.1186/s13750-018-0138-y”	DOI, citation, or URL for the protocol or registered report outlining the planned search methods	Optional	
Linked final review document	“10.5438/55e-987-a5-t5c0”	DOI, citation, or URL for the final review document describing the search methods used	Optional	

*Note*: See Supporting Information: Files 2 and 3 for further examples of other forms of searches.

Here, we justify the inclusion and formatting of each item:

1. Authors—due to the flexibility of a JSON format, each field may contain nested structured data. Here, the author field can contain author name, ORCID identifier and email addresses for each author. This field corresponds to authors of the search strategy and is intended to provide acknowledgement and credit to search specialists. Authorship should be decided based on clearly defined and widely accepted definitions of co‐authorship, for example, by adapting the CRediT authorship statement from the high‐profit publisher Elsevier (https://www.elsevier.com/authors/policies-and-guidelines/credit-author-statement). Authorship on a search record (i.e., a record that documents a specific search history) should not be used as justification for removing a search specialist from a review: such behaviour would be unethical at best.
2. Dates—data entry date refers to the date the search history item was created (this should be created automatically by any platform); strategy export date refers to the date the search strategy was exported (if different to the conduct date and most relevant where search history was exported and entered automatically from bibliographic databases); search conduct date refers to the date the search was performed and results exported; search update date(s) refer to any dates that the search was repeated to capture additional information.
3. String name—this is an optional ‘tag’ for internal purposes, for example labelling a substring as ‘intervention’ or ‘outcome’.
4. Keywords—these are additional terms that may be useful in searching for the search strategy. Searching across search strategies should of course focus on search strings, but keywords here are an optional record‐level means of increasing discoverability. For ease, these could be keywords from relevant related publications that describe the searching (e.g., a systematic review protocol).
5. Related search records—this field records the DOIs for related searches documented using a DOI, for example, searches of different databases for the same systematic review.
6. Overview entry for this substring—this field provides the DOI for the overview record, if present. Here, a substring is defined as a string that is combined with other strings to form a single search within a given resource. The overview record clusters searches that have been used for the same project so they are more easily linked.
7. Platform—this refers to the system used to interrogate the database by means of searching. The platform dictates the search functionality used in the search and the format and settings of the search strategy. A single database may be searched in different ways via different platforms (e.g., MEDLINE via Web of Science or PubMed).
8. Database—this refers to the index containing bibliographic data (e.g., MEDLINE or CAB Abstracts). In theory, different users searching a database using identical searches at the same moment should find identical search results.
9. Search string (also referred to sometimes as the ‘search strategy’)—this refers to the text entered into the search function on the platform, copied and pasted precisely. For some platforms this will be multi‐line searches, for others it will be complete blocks of search terms. Some strings will include field terms and additional settings, whilst for other platforms the fields searched and settings may be manually specified and described below in further optional fields.
10. Search fields—where search strings are entered into search functions separately from specifying the search field, users should specify the search fields here (e.g., TS/topic words for Web of Science platform searches).
11. Database time coverage from/to—these fields refer to the date ranges covered by the databases on the day the search was conducted. Often these are full years (e.g., 1975‐present), but may not be known or easily discoverable; hence this item is optional. Date ranges for databases can be important since some platforms provide different periods of coverage to different subscribers (e.g., the Science Citation Index Expanded provided via Web of Science).
12. Date limitations—this refers to optional date ranges specified within the search, restricting the results to a given period, not already specified in the search string itself. Depending on the database this may either be the indexing date (the date on which the record was indexed in the database) or, more commonly, the publication date.
13. Language—this refers to the optional specification of record language that may be specified when searching, not already specified in the search string itself.
14. Settings—this field contains any other optional settings (e.g., lemmatisation or term expansion) not already specified in the search string itself.
15. Quality assurance—this refers to the type of quality assurance provided to the search strategy and may be one of the following: “exploratory search” (used to develop a search strategy iteratively), “appears in published protocol” (indicating the planned search has likely undergone some form of peer‐review), “appears in published review” (indicating the enacted search has likely undergone some form of peer‐review), “peer‐reviewed” (some other form of peer‐review has been conducted), “validated against benchmark articles” (indicating that the search has been validated against a set of predefined records of known relevance). Multiple values may be chosen.
16. Validation report—this field can indicate whether the search has undergone formal research validation, meaning that the specificity and sensitivity have been formally assessed. See Durão et al (Durão et al., 2015). for an example of a search string validation.
17. Description—this field should be used to give a short textual description of the search string/strategy, and may take the form of the abstract from the review protocol or final review of which the search was part. Authors may also wish to describe the context and/or possible limitations of the search, for example that the search aimed to maximise specificity (precision) at the expense of sensitivity (recall) because of resource constraints.
18. Review question—this refers to the overall primary question for the associated review.
19. Review type—this is an optional field relevant to searches that are undertaken as part of a full literature review and should be one of the following: “systematic review”, “systematic map”, “scoping review”, “rapid review”, “other literature review”
20. Linked review protocol/registered report—where searches are undertaken as part of a published review, this field can be used to link to the published protocol (preprints included).
21. Linked final review document—where searches are undertaken as part of a published review, this field can be used to link to the published final review report (preprints included).

### 3.2 Suggested file format

The data structure proposed above could be encoded within a standardised filetype, for example, a JavaScript object Notation (JSON) file. JSONs lend themselves well to this form of data structure for several reasons, including that: these files are specifically designed for transmitting information between softwares and over the internet; the file contents are coding language independent, self‐explanatory, and readily understandable by human and machine (Wehner et al., 2014); data structures are nested and hierarchical, meaning that a single field can contain further two‐dimensional datasets (e.g., a single field labelled ‘authors’ can contain multiple sub‐fields for ‘author names’, ‘emails’ and ‘affiliations’).

In a JSON file, data are represented as ‘name‐value’ pairs (e.g., “email”: “neal_haddaway@hotmail.com”); names are indicated by a colon suffix (e.g., “email”:); ‘objects’ are held within curly brackets (‘{}’); arrays of multiple values for a single name are comma‐separated and held within square brackets (e.g., “email”: [“neal_haddaway@hotmail.com”, “neal.haddaway@sei.org”]).

A proposal for a data structure for a JSON file for search histories needs only contain a set of standard field labels and specification of which fields contain subfields (nested data). We suggest this structure in Table 1 and provide an example file text in JSON format in Box 1. If these labels and structure were to be adopted across platforms and software, search histories could be shared and reused digitally without impediment.

Box 1. **Example JSON file format for standardised search strategy data. ‘\’ is an escape sequence used to remove coding functionality (“would otherwise denote the end of the text string) from quotations in string (text) fields, and can be automatically added to plain text programmatically**

**Table:** 

{
“record_info”: {
“id_1”: “10.1897/687-asdg9-88.10”,
“repository”: “SearchRxiv, https://www.searchrxiv.org”,
“internal_id”: “10.1897/687-asdg9-88.10”
},
“authors”: [
{
“name”: “Seedre, M”,
“ORCID”: “0000‐0002‐3635‐6354”,
“email”: “m.seedre@mail.com”
},
{
“name”: “Felton, A”
},
{
“name”: “Lindbladh, M”
}],
“date”: {
“data_entry”: “2021.03.02”,
“strategy_exported”: “2021.03.02”,
“search_conducted”: “2020.01.01”,
“search_updated_1”: “2021.02.01”
},
“string_name”: “final”,
“keywords”: [“Alternative forest management”, “Even‐aged silviculture”, “Uneven‐aged silviculture”, “Partial harvest”, “Selection harvest”, “Clear cut”, “Clear fell”],
“related_records”: “10.5438/67dgh-t5666oop”,
“parent_record”: “10.5438/67dgh-t56-78p”,
“platform”: “Web of Science”,
“database”: [“Social Sciences Citation Index (SSCI)”],
“search_string”: {
“main”: “TS=(clear‐cut* OR clearcut* OR clearfell* OR clear‐fell* OR\“clear fell*\“ OR even‐aged OR uneven‐aged) AND (forest* OR tree*)”,
“management block”: “TS=(clear‐cut* OR clearcut* OR clearfell* OR clear‐fell* OR\“clear fell*\“OR even‐aged OR uneven‐aged)”,
“forest block”: “TS=(forest* OR tree*)”
},
“search_field”: null,
“database_time_coverage”: {
“database_time_from”: “1945.01.01”,
“database_time_to”: “2021.12.31”
},
“search_time_span”: null,
“search_language”: [“en”],
“settings”: {
“lemmatization”: “TRUE”,
“spellchecking”: “Suggest”
},
“quality_assurance”: [“appears in published protocol”],
“validation_report”: [“10.5438/67dgh-t56-78b”],
“peer_review”: [“https://pubpeer.com/publications/3C0A753937515D4ACAEDB31CAE22C2”],
“description”: “The search was conducted as part of a systematic review, developed during the publication of a protocol ([link]) and finalised during conduct of a systematic review ([link])”,
“review_question”: “What is the impact of continuous cover forestry compared to clearcut forestry on stand‐level biodiversity in boreal and temperate forests?”,
“review_type”: “systematic review”,
“linked_protocol”: [“10.1186/s13750-018-0138-y”],
“linked_report”: [“10.5438/55e-987-a5-t5c0”]

The use of this JSON structure goes beyond basic tool interoperability by using a common language by allowing information to be embedded within the bibliographic data file itself. The JSON file could be embedded within a bibliographic data field, for example within the ‘DB’ (database) field in the first record in an RIS file. This would allow each search result file to be ‘tagged’ with the search history for that set of results. Alternatively, the DOI (digital object identifier) of the search history record in a suitable repository (e.g., www.searchrxiv.org) could be added alone in a single field. The full search history could then be extracted (automatically, if desired) by following this DOI as a URL. This may be particularly useful for retaining record‐level meta‐data regarding the sources of records throughout a review. Where deduplication removes duplicates, a review management tool could append multiple source DOIs as an array or comma‐separated list.

We have built a prototype web‐based tool for embedding search data within an RIS file: https://estech.shinyapps.io/searchrecorder/. Users can embed the example JSON file, or edit the file themselves.

## 4 CONCLUSIONS

Searching for information is arguably the most important step of any evidence synthesis, since it must be conducted in a way so as to maximise comprehensiveness and minimise bias in the returned set of final search results (Rethlefsen et al., 2021). To be sure that searches have been performed correctly, it is necessary for review authors to accurately and completely report their search activities. Bibliographic database searching is a cornerstone in the vast majority of evidence syntheses, contributing the majority of evidence in most reviews (Haddaway & Westgate, 2019). To date, however, most evidence syntheses do not report their searches in sufficient detail to allow repeatability or evaluation (Abbott et al., 2022; de Kock et al., 2021; Koffel & Rethlefsen, 2016; Maggio et al., 2011; Mullins et al., 2014; Yoshii et al., 2009). Therefore, there is clearly a need for efforts to improve the reporting of evidence synthesis search strategies, particularly for bibliographic database searching.

Reporting standards for evidence syntheses are necessary and important, but alone may be insufficient to encourage authors to report searches in a fully transparent and repeatable manner. Given the rapid increase in publication of evidence syntheses (demonstrated by the recent explosion of systematic reviews on covid‐19: (Abbott et al., 2022; Dotto et al., 2021), there is an urgent need to rapidly improve reporting of these reviews.

As tools develop, novel access points show promise in increasing the transparency and replicability of searches—namely, APIs (application programming interfaces), which allow a query to be sent and the results from a database/platform search to be received within a programme or software (e.g., Scopus API in R (Muschelli, 2019)). However, given that many platforms provide different contents depending on subscription, and that this information is not coded within an API query, an API code is insufficiently transparent and replicable alone—a data standard is still required.

Here, we have proposed a set of fields and a proposed file type for reporting bibliographic search strategies in a transparent and repeatable manner. We believe this standard will support: greater transparency and repeatability; a reduction in typographical errors; greater, more efficient and more accurate reuse of search strategies; development of repositories of search strategies for clearer sharing and crediting of searches; learning and awareness raising about the nuance of search strategy reporting; and, better acknowledgement and crediting of search specialists.

We hope that developers of review management tools and search strategy repositories will employ this data structure to assist in transparent reporting of search histories by their users. We suggest the standardised data file in JSON format may be a useful interoperable format to employ. We encourage the community to extend and develop the data standard as necessary. We hope that keen systematic reviewers may also use the data structure already in their own search strategy reporting where they do not have a suitable repository of review management tool. We believe adoption of this standard by tool developers would be a modest investment in the rigour of systematic reviews broadly. Such integration in easy‐to‐use and particularly Open Source tools and repositories would allow users to report searches transparently with minimum effort. Furthermore, we would hope that a search history repository (such as www.searchrxiv.org) that properly gave credit for the hard work of librarians in designing and conducting searches would drastically reduce barriers to uptake of this data structure.

Finally, we call for further discussion of these data standards and adoption of a standardised file type for ensuring transparency, consistency, and interoperability of academic search histories.

## Supporting information

Supporting Information 1.

Supporting Information 2.

Supporting Information 3.
